# To be or not to be an inclusive teacher: Are empathy and social dominance relevant factors to positive attitudes towards inclusive education?

**DOI:** 10.1371/journal.pone.0225993

**Published:** 2019-12-10

**Authors:** Diego Navarro-Mateu, Jacqueline Franco-Ochoa, Selene Valero-Moreno, Vicente Prado-Gascó

**Affiliations:** 1 Department of Educational Psychology and Special Educational Needs, Faculty of Psychology, Teaching and Educational Sciences at the Catholic University of Valencia, Valencia, Spain; 2 Faculty of Psychology, Teaching and Educational Sciences at the Catholic University of Valencia, Valencia, Spain; 3 Department of Personality, Assessment and Psychological Treatments, Faculty of Psychology, University of Valencia, Valencia, Spain; 4 Department of Social Psychology, Faculty of Psychology, University of Valencia, Valencia, Spain; Universidade de Mogi das Cruzes, BRAZIL

## Abstract

Positive inclusive teacher attitudes are a key factor in achieving inclusive education due to the many benefits they generate for schools and social contexts. Studies have focused on analysing which variables may promote positive attitudes. The objective of this study was to analyse the predictive power of sociodemographic variables, empathy (cognitive and emotional), and social dominance orientation (social dominance and opposition to equality) on teachers’ attitudes, sentiments, and concerns about inclusion by comparing linear relationship models and models based on fuzzy-set comparative qualitative analysis. The sample consisted of 268 teachers of different educational levels aged between 20 and 64 years (M = 42.46, SD = 9.22), 66% of whom were women. The teachers were administered the Sentiments, Attitudes, and Concerns about Inclusive Education Revised (SACIE-R) scale, the Basic Empathy Scale (BES), and the Social Dominance Orientation (SDO) scale. Two different statistical methodologies were used: traditional regression models and fuzzy-set qualitative comparative analysis (QCA) models. The results of the regression models suggest that social dominance is the main predictor of attitudes, sentiments, and concerns about inclusion. Social dominance is negatively related to attitudes and positively related to sentiments and concerns. Only opposition to equality and emotional empathy are related to attitudes. On the other hand, the results of the QCA models suggest that low social dominance and high cognitive and emotional empathy, as well as female sex, predict higher levels of positive attitudes, sentiments, and concerns about inclusion. Since teachers interact most with students, it is important to be aware of how their empathy and social dominance orientations influence inclusion to develop intervention programmes that seek to train teachers in these capabilities.

## Introduction

The purpose of inclusive education is to provide educational attention that favours the maximum possible development of all students and the cohesion of all members of the community[1]. For this reason, the majority of the world’s countries have proposed to guarantee inclusive and quality education for all by 2030, thus reducing the negative impact on the emotional well-being of students and improving coexistence in classrooms[2]. However, there is high variability in the implementation of inclusive education, which is reflected in both regional and school-specific differences[3]. In Spain, the legislative milestones related to inclusive education are the development of the General Education Law in 1970, the Organic Law on the General Organization of the Educational System in 1990, the Organic Law on Education in 2006 and the current Organic Law for the Improvement of Educational Quality in 2013. Despite progress in terms of access to mainstream schools for excluded students, the achievement of legitimate inclusive education is still in process. Rather than making a declaration of inclusive intentions, which are incoherent with educational policy and practice in many cases, the true meaning of inclusive education is still in the process of being constructed and integrated into practice. Thus, a change of vision is required that involves a change of perspective to understand that every student should be accepted and valued as a human being with full rights and in terms of his or her own diversity[4].

Inclusion and an adequate response to diversity are objectives that education professionals must continuously pursue, with special attention to children who are most vulnerable and at risk of exclusion[5].

From this perspective, it is of particular interest to be able to understand what aspects may lead to the better inclusion of people with special needs in the classroom. Among the factors that can influence this situation, the behaviour of teachers is especially important[5]. Therefore, it seems necessary to deepen the understanding of the reasons or explanations for the behaviour of these professionals.

In this respect, as the theory of planned behaviour (PB)[6] suggests, individuals’ attitudes represent the best predictor of their behavioural intentions. On the other hand, according to the aggression model, beliefs about the type of relationship and interaction between different groups lead to relationships of either domination or equality[7]. Stereotypes, prejudices, and discriminatory behaviour are three powerful and deeply interrelated components that can influence inclusion in the school context, as various studies have demonstrated[8,9].

PB theory clarifies the decision-making process and its relation to behaviour in complex contexts, such as education. Thus, it can help us to better understand teachers’ behaviour. Based on this theory, it is suggested that in addition to the attitudes of the subject (in our case, teachers), other factors intervene, such as subjective norms (social norms) and the subject’s perceived behavioural control (beliefs and subjective evaluation of one’s own capacities to perform a given action). In our context, there seems to be a widespread social norm that promotes the need for inclusion in the classroom and therefore facilitates behaviours in this sense[4]. In addition, teachers have control over the actions to be performed in the classroom when interacting with people with specific needs (behavioural control). Such behavioural control may be influenced by teachers’ competencies or skills, such as empathy[10,11]. For this reason, given that both social norms and behavioural control promote inclusive behaviours, teachers’ attitudes towards inclusive education seems to be what mainly determines whether teachers perform behaviours that promote inclusion in the classroom. Attitudes can also be influenced by a variety of factors, including other attitudes, such as teachers’ attitudes towards intergroup relationships[6,7,8].

Within this framework, teachers’ positive attitudes towards inclusion are a key factor in the success of school inclusion[12]. In this sense, the type of response (i.e., positive or negative) that a teacher has[5] towards a specific issue, such as school diversity, will depend on the combination of three integrated components of attitude: the cognitive component (positive or negative beliefs towards diversity), which is equivalent to the concerns component; the affective component (positive or negative evaluations of diversity), which is similar to the feelings dimension that translates into acceptance (inclusion) or rejection (exclusion); and the conative-behavioural response (disposition towards diversity in relation to the other two components), which is the most attitudinal component[13].

Several studies have shown that teachers’ attitudes and beliefs seem to positively affect inclusive education in the classroom[14–17]. In some cases, negative attitudes, beliefs, and prejudices accompanied by discriminatory and exclusionary actions towards stigmatised groups, such as people with disabilities, have been reported[18,19]. In this regard, it seems that beliefs and attitudes towards diversity can facilitate or hinder inclusion in the educational context[20,21].

Therefore, positive attitudes towards diversity and inclusive teaching skills are two basic characteristics in the teaching profession[22]. Competencies are sets of knowledge, skills, abilities and attitudes that are necessary to carry out activities with a certain degree of quality and effectiveness and that integrate the aspects of knowledge, know-how, and knowing how to act[23]. Among the different competencies that are required to be a good teacher, the European Agency[1] highlights empathy, arguing that for the development of inclusive contexts, it is necessary to “show sufficient empathy with the diverse needs of students”[1] Thus, a teacher’s empathy, understood as the ability to put oneself in the place of the other (cognitive empathy) and to share one’s emotions (emotional empathy) [24], is considered a key factor in the educational context[25], particularly in relation to people belonging to stigmatised groups or those at risk of exclusion[11,26]. Empathy can increase understanding and knowledge about the circumstances of such people[26], which in turn affects attitudes towards them[27] and, ultimately, attitudes towards educational inclusion. Cultivating empathy towards vulnerable or marginalised groups, such as students with special needs, improves attitudes towards these groups[28] while increasing positive behaviours towards them[26]. Empathy can optimise inclusive attitudes and interpersonal and inter-group relationships[28,29] and reduce prejudices based on negative stereotypes and erroneous messages about groups that are stigmatised or at risk of exclusion[30].

As with empathy, beliefs about intergroup relationships have been found to be one of the best predictors of prejudice and discrimination[31] and thus one of the greatest barriers to teachers’ inclusive attitudes. Social organisation is usually established due to the existence of social groups[32] with a shared system of beliefs, values, interests, and symbols, which leads to the recognition of differences between the group to which an individual belongs (*us*) and the out-group (*them)*[33]. Thus, based on the theory of self-categorisation[34], comparison between the in-group (the dominant group) and the out-group (the group of people with disabilities) increases the differences between the two, which results in the creation of stereotypes shared by the members of the endo-group (the dominant group). This distinction leads to different attitudes towards the in-group and the out-group[35,36]. The type of relationship and interaction with the external group give rise to cooperative or competitive relationships, collaboration, or conflict—the latter being the most common result[7]. In this regard, Tajfel[33] argues that the dominant group promotes motivation and in-group favouritism while discriminating against the out-group. Thus, some groups are established as superior or dominant, which translates into increased power, social status, political control, and a predisposition to maintain control over access to social resources and opportunities[31,37], while other groups, such as disadvantaged groups or people with special educational needs, are established as inferior. Based on the theory of social dominance, Pratto et al. seek to explain the processes that produce and maintain social hierarchies. A social dominance orientation (SDO) would explain a desire to maintain the social hierarchy and inequality towards the group or collective of people with disabilities[31,38], which is defined and categorised as inferior through an extensive language of labels that define them as *students with special educational needs* and contrast them with students in the majority, superior group, who are considered *normal* and without *needs*[21]. Thus, high levels of SDO promote unequal and hierarchical intergroup relations[31]. High levels of SDO are also associated with negative attitudes towards different minority groups at risk of exclusion[39,40]. In this way, an SDO towards attitudes related to inclusion explains how the configuration of negative stereotypes—the belief that difference and diversity are abnormal—results in the harmful effect of categorising and classifying students with disabilities[41]. This, in turn, acts as the basis for the justification of prejudices and discriminatory and exclusionary actions towards these students[42]. In short, the effect of the dominant perspective on education contributes to the rejection and exclusion of certain students[43]. This is certainly contrary to the inclusive perspective, which is based on the core concept that students have the right to be different and that diversity is normal[5].

Despite the importance of the processes of justification of intergroup relations and, more specifically, of an SDO towards inclusive attitudes, we have been unable to find any study that has analysed their effects on active teachers’ attitudes towards inclusion. Likewise, very few studies have analysed the effect of empathy on active teachers’ attitudes towards inclusion, especially in the Spanish context. We have not been able to identify any study that has simultaneously considered the influences of empathy and SDO on teachers’ inclusive attitudes.

According to Van Mieghem’s review[44], there are other variables that influence attitudes towards inclusion. On the part of teachers, teaching experience in inclusive education issues, perceived self-efficacy, and the type of need of the student (whether it is an emotional disorder or severe cognitive impairment) affect attitudes towards inclusion.

Other factors specific to teachers that also seem to be related to teachers’ attitudes towards inclusion include age, sex, and experience and contact with people with special educational needs[13,44–47]. In this sense, the literature suggests that experience with people with special educational needs positively affects teachers’ inclusive attitudes[19,26,30] as this experience may increase their confidence[13,47]. Likewise, although there seems to be some agreement on the existence of differences in the roles and socialisation processes of men and women in relation to teachers’ attitudes towards inclusive education[48], the results regarding the influence of sex on attitudes towards inclusion remain less clear. Much of the literature suggests that female teachers have more positive attitudes towards inclusion[45], while other studies have not observed differences between male and female teachers[49] or have even suggested more favourable attitudes among male teachers[50]. Finally, with respect to age, several studies have suggested that younger teachers tend to adopt more favourable views towards the inclusion of students with special educational needs[13,45], while some older teachers appear to tend towards the opposite stance[51]. Previous studies have analysed how some of these aspects influence inclusive education in isolation[7,8,26] but not as a whole.

Similarly, most of the available studies in the area of psychology have focused on methodologies based on linear models[52,53], ignoring other methodologies based on non-linear relationships. These linear models have examined the individual contribution of each of the variables; however, they have not considered a priori combinations of different study variables, nor have they taken equifinality into account[54,55]. QCA is an analytical technique that allows in-depth analysis of how a series of causal conditions contribute to a particular result[56–58]. QCA models are based on Boolean logic, and the results depend to a large extent on combinations of attributes rather than on the individual contribution of each attribute[59].

Given the importance of the development of teachers’ inclusive attitudes, the influences of empathy, SDO, and sociodemographic factors such as age, sex, or experience on these attitudes, the lack of studies that have considered SDO or that have simultaneously analysed all of these variables, and the scarcity of literature in psychology that combines linear models with models based on QCA, the significance of the present study is clear. This study aims to analyse the impact of empathy, SDO, and sociodemographic variables on teachers’ attitudes through hierarchal regression models (HRMs) and QCA models.

Based on the literature consulted, the following hypotheses were formulated:

H1: Social dominance negatively influences attitudes towards inclusion but, in contrast, will positively influence concerns, sentiments, and opposition to equality.
H2: Empathy is positively related to attitudes towards inclusive education and negatively related to concerns and sentiments.
H3: Work experience is positively related to attitudes towards inclusive education and negatively related to concerns and sentiments.
H4. Being a woman positively influences attitudes towards educational inclusion and negatively affects concerns and sentiments.
H5. Age is negatively related to attitudes towards educational inclusion and positively related to concerns and sentiments.

## Method

### Participants

A total of 268 teachers between the ages of 20 and 64 participated in the study (M = 42.46 years, *SD* = 9.22). Thirty-four percent were men, and 66% were women. They had an average of 17 (SD = 9.21) years of teaching experience, and their years of experience ranged from 0 to 39 years. Regarding the educational levels the teachers taught, 29% taught infant education, 73.1% taught primary education, 19.4% taught secondary education, and 7.1% taught high school. Finally, regarding the frequency with which teachers had taught students with functional diversity, 40.44% often or very frequently taught these students, 32.29% indicated that they had sometimes taught these students, and 27.27% indicated that they had never or rarely taught students with disabilities.

### Instruments

#### Spanish adaptation of the Sentiments, Attitudes, and Concerns about Inclusive Education Revised (SACIE-R)[13]

The SACIE-R scale was adapted according to the guidelines of the International Testing Commission (ITC)[60]. For the adaptation of the instrument to the linguistic context, two native linguists translated and back-translated (English-Spanish-English) the items of the scale. This process was complemented by a review of the resulting items by experts in inclusive education, which ensured the adequacy of the statements for the Spanish context. The scale is composed of 15 items with a Likert-type response format with five anchors (1 = *strongly disagree*, 2 = *disagree*, 3 = *neither agree nor disagree*, 4 = *agree*, 5 = *strongly agree*). Five items refer to affective factors (e.g., “Students who have difficulty expressing their thoughts verbally should be in ordinary classrooms”); five items refer to attitudes (e.g., “Students who need personal care assistance should be in ordinary classrooms”); and five items refer to concerns (e.g., “I am concerned that if I have students with disabilities in my class, my workload will increase”). Overall, the scale has demonstrated good reliability in previous studies[46]. In the present study, the scale also showed adequate psychometric properties: χ^2^(df) = 188.02(41); SB-χ2(df) = 157.26(41); χ2/df = 4.58; CFI = .87; IFI = .87; RMSEA = .094 (IC = .078–.109). In the present study, reliability values of .84 (CI = .81-.87), .64 (CI = .57-.70) and .61 (CI = .53-.68) were found for attitudes, sentiments and concerns, respectively.

#### Basic Empathy Scale (BES)[61]

This scale has 20 items that evaluate cognitive empathy and emotional empathy. Specifically, nine items assess cognitive empathy, and 11 items assess emotional empathy. Respondents indicate their responses on a Likert scale with five anchors (1 = *strongly disagree*, 2 = *disagree*, 3 = *neither agree nor disagree*, 4 = *agree*, 5 = *strongly agree*). Eleven items refer to emotional empathy (e.g., “I don’t feel anything when my friends are unhappy”), and nine items refer to cognitive empathy (e.g., “I understand the happiness of my friends when something happens to them”). Because the original model, which was composed of 20 items in two dimensions, did not present a good fit for the sample as a whole, several re-specifications were made. Several items with high residues (>.20) were eliminated, yielding a final model that showed a good fit. This final model was composed of 9 items distributed in two dimensions: emotional empathy (4 items) and cognitive empathy (5 items).

The scale has demonstrated adequate psychometric properties in previous studies[57], and adequate psychometric properties were also observed in the present study: χ2(df) = 86.04(26); SB-χ2(df) = 112.76(26); χ2/df = 3.31; CFI = .83; IFI = .84; RMSEA = .08 (IC = .065-.104). In the present study, a reliability of .55 (CI = .46-.62) was obtained for emotional empathy, and a reliability of .66 (CI = .60-.71) was obtained for cognitive empathy.

#### Spanish adaptation[62] of the SDO (SDO) scale[31]

This questionnaire enables the evaluation of the psychological mechanism known as social dominance orientation. Its purpose is to identify the desire to establish and maintain social hierarchies involving inequality and the subordination of certain groups that are classified as inferior. The scale comprises 16 statements that refer to hierarchical relationships between groups within society and consists of two factors: opposition to equality and group dominance (e.g., We would have fewer problems if we treated different groups more equally). For this study, the adaptation of the SDO scale by Etchezahar[63] was used. The items are answered on a Likert scale with five response anchors ranging from 1 = *strongly disagree* to 5 = *strongly agree*, as for the other two scales used in this study. The scale has demonstrated adequate psychometric properties in previous studies[58–59], and adequate psychometric properties were also observed in the present study without any re-specification: χ2(df) = 96.43(34); SB-χ2(df) = 77.07(34); χ2/df = 2.83; CFI = .92; IFI = .92; RMSEA = .063 (IC = .044-.081). In the present study, a reliability of .71 (CI = .66-.76) was obtained for social dominance, and a reliability of .66 (CI = .60-.71) was obtained for opposition to equality.

### Procedure

This study adhered to the fundamental principles of the Declaration of Helsinki, with particular emphasis on the anonymisation of the data collected, confidentiality, and non-discrimination of the participants. Due to the nature of the study, the anonymity of the questionnaires and the voluntary nature of participation, the bioethics committee of the Catholic University of Valencia stated that the approval of the ethics board was not necessary. All participants were of legal age and voluntarily agreed to participate in the study. Data collection was conducted over a period of four months in 2017. After schools in the Valencian Community were selected, they were contacted, and the project was explained to school staff. Subsequently, sessions were organised with the teachers who wanted to participate to inform them about the project, the benefits of participation, and the implications of participating in the study, which would involve voluntarily completing an anonymous questionnaire. The schools that expressed a desire to participate were provided with the total number of questionnaires requested according to the number of teachers who voluntarily agreed to participate along with sealed ballot boxes in which the questionnaire could be deposited to guarantee the anonymity of the participating subjects.

### Data analysis

First, descriptive analyses of the participants were conducted, and calibration values for QCA were calculated. Then, Pearson’s correlations, hierarchical regression modelling and QCA were performed. Regarding the hierarchical regression models, three models with three steps that considered each of the dimensions of the SACIE-R were calculated.

To develop the QCA models, the raw data from the participants’ responses were transformed into fuzzy-set responses. First, as suggested in the literature, all missing data were deleted, and all constructs (variables) were calculated by multiplying their item scores[54,55,64]. Before performing the analysis, the values had to be recalibrated between 0 and 1. Recalibration is very important because it can affect the final result by indicating that more or fewer observations or participants had a certain result. When only two values are considered, 0 (which indicates the absence of the characteristic of interest and thus that the observation is completely outside the set) and 1 (which indicates the presence of the characteristic and that the observation is completely inside the set) are used. However, to perform the recalibration with more than two values, the following three thresholds are used: the first value (0) indicates that an observation is completely outside the set (a low level of agreement); the second value (0.5) is a midpoint indicating that the observation is neither inside nor outside the set (an intermediate level of agreement); and the last value (1) indicates that the observation is fully inside the set (a high level of agreement). This process is the direct method of calibration proposed by the author of the methodology, Ragin[58], which is most commonly used in the literature[65–68]. With continuous variables or with questionnaire dimensions (formed from different items), the three aforementioned values are used. To achieve an automatic recalibration of values between 0 and 1, the literature suggests using three thresholds: the 10th, 50th, and 90th percentiles[69]. The QCA 2.5 software by Claude and Christopher[70] was used to recalibrate the SDO values, cognitive and emotional empathy values, and attitudes, concerns, and sentiments values from the SACIE-R considering the following three thresholds[69]: 10% (low agreement, or fully outside the set), 50% (intermediate level of agreement, neither inside nor outside the set), and 90% (high agreement, or fully inside the set). Once the responses were transformed, as suggested by the literature, *necessary* and *sufficient* condition tests were used to evaluate the effect of the sociodemographic and work experience variables, social dominance, and empathy on a particular outcome (inclusive education, measured by means of the three SACIE-R dimensions) and on the absence of the output (the absence of inclusive education). A *sufficient* condition exists when there is a combination of conditions that can produce a particular outcome, but that particular outcome could also be achieved by other combinations of conditions. Conversely, a condition is *necessary* when it must always be present for the occurrence of a particular outcome. According to Eng and Woodside, calculating sufficient conditions with QCA involves two stages[56]: first, a truth table algorithm is used to transform the fuzzy-set membership scores into a truth table that lists all logically possible combinations of causal conditions and the empirical outcome of each configuration; second, QCA generates three possible solutions, i.e., complex, parsimonious, and intermediate. The complex solution is the most restrictive, and the parsimonious solution is the least restrictive. Previous studies[58] have suggested including an intermediate solution (the solution that is presented here). In a sufficient condition analysis, as stated above, the solution coverage considers the explained variance (the number of observations that can be explained by a particular combination of conditions), whereas the solution consistency expresses a model’s possible reliability or fit. In addition, when considering each condition, raw coverage indicates how many cases or observations can be explained by the conditions (explained variance). Conversely, the unique coverage expresses the number of observations (variance) that can be explained by a particular combination of conditions but not by other combinations of conditions. To choose the most important condition, the raw coverage must be considered. With regard to the similarity between *necessary* and *sufficient* condition analyses, *consistency* indicates the adequacy of the condition to predict a particular outcome (≥.90), whereas *coverage* considers the variance explained by a condition[58]. SPSS (Statistical Package for the Social Sciences, Version 23, ©IBM) was used to perform the descriptive analysis, calculate the calibration values, and develop the HRMs, and QCA software (fuzzy qualitative comparative analysis, version 2.5, ©Raging and David, 1999–2008[70] was used to perform the QCA.

## Results

First, the main descriptors and calibration values for the variables under study are presented (Table 1).

**Table 1:** Main descriptions and calibration values.

	Sociodemographic variables		SDO^a^		BES^b^		SACIE-R^c^		
	Age	Teaching experience	Social dominance	Opposition to equality	Cognitive empathy	Emotional empathy	Attitudes	Concerns	Sentiments
*M*^d^	42.46	17	16.56	1821.90	936.50	217.08	1472.34	29.48	26.91
*SD*^e^	9.22	9.21	36.01	996.77	698.52	160.90	1022.84	27.86	24.37
*M*_*according to the average values of the scales*_	-	-	1.51	4.41	3.81	3.71	4.08	2.91	2.94
*SD*_*according to the average values of the scales*_	-	-	0.54	0.54	0.59	0.70	0.73	0.88	0.88
*Min*^f^	20	0	1	72	1875	4	9	1	1
*Max*^g^	64	39	324	3125	9765625	625	3125	125	125
		*Calibration values*							
*P10*^h^	32.9	5	1	448	216	48	265	4	4
*P50*^i^	41	15	3	1875	768	192	1225	18	18
*P90*^j^	55.10	30	48	3125	2000	500	3125	60	75

^a^ scale of orientation to social dominance

^b^ basic scale of empathy

^c^ Sentiments, Attitudes, & Concerns about Inclusive Education Revised

^d^ mean

^e^ standard deviation

^f^ minimum

^g^ maximum

^h^ 10th percentile

^i^ 50th percentile

^j^ 90th percentile

### Hierarchal regression models (HRMs)

The relationships between variables were first analysed with Pearson correlations and then with hierarchical regression models.

Based on the correlations, attitudes towards inclusion were found to be positively related to emotional empathy and opposition to equality and negatively related to social dominance *(p≤* .*001)*. Sentiments and concerns about inclusion were negatively related to social dominance *(p≤* .*001 and p≤* .*01)*, and concerns were positively related to emotional empathy *(p≤* .*01)* (Table 2).

**Table 2:** Pearson’s correlations with SACIE-R, SDO, BES and sociodemographic variables.

	1	2	3	4	5	6	7	8	9
1. Age	1								
2. Years of experience	.92***	1							
3. Social dominance	.-15*	-.12*	1						
4. Opposition to equality	.13*	.07	-.29***	1					
5. Emotional empathy	-.16**	-.09	-.09	.09	1				
6. Cognitive empathy	-.03	.03	.01	-.01	.40***	1			
7. Attitudes	-.04	-.06	-.24***	.27***	.21***	.04	1		
8. Sentiments	.03	.05	-.21***	-.06	-.04	.01	-.32***	1	
9. Concerns	-.09	-.13*	.19**	-.10	-.04	-.10	-.15**	.29***	1

*** p≤.001;

** p≤.01;

*p≤.05

The predictive power of the variables under study was then analysed using hierarchical regression models (Table 3). The criterion variables used were attitudes, sentiments, and concerns from the SACIE-R. The predictor variables were sociodemographic variables (sex and age), years of experience, SDO (social dominance and opposition to equality), and empathy (cognitive and emotional). Each model involved three steps: first, sociodemographic variables and years of experience were included; second, the variables of social dominance and opposition to equality (SDO scale) were introduced; and finally, cognitive and emotional empathy (BES) were added.

**Table 3:** Hierarchical multiple linear regression models for inclusive education dimensions (SACIE-R).

Variable	Attitudes				Sentiments				Concerns		
		ΔR^2^^a^	β^b^			ΔR^2^	β			ΔR^2^	β
**Step 1**		.01		**Step 1**		.01		**Step 1**		.03	
	Sex		.03		Sex		-.04		Sex		.08
	Age		.14		Age		-.16		Age		.19
	Teaching experience		-.18		Teaching experience		.19		Teaching experience		-.29
**Step 2**		.11***		**Step 2**		.05***		**Step 2**			
	Sex		.01		Sex		-.04		Sex	.03**	.08
	Age		-.01		Age		-.12		Age		.22
	Teaching experience		-.09		Teaching experience		-.18		Teaching experience		-.30*
	Social dominance		-.20**		Social dominance		.23***		Social dominance		.19***
	Opposition to equality		.22***		Opposition to equality		.01		Opposition to equality		.03
**Step 3**		.03**		**Step 3**		.001		**Step 3**		.01	
	Sex		-.04		Sex		-.03		Sex		.09
	Age		.06		Age		-.12		Age		.19
	Teaching experience		-.13		Teaching experience		.18		Teaching experience		-.27
	Social dominance		-.18**		Social dominance		.22***		Social dominance		.19***
	Opposition to equality		.21***		Opposition to equality		.01		Opposition to equality		.03
	Cognitive empathy		-.03		Cognitive empathy		.01		Cognitive empathy		-.09
	Emotional empathy		.19**		Emotional empathy		.03		Emotional empathy		.01
**Total R**^**2**^**adj**^c^ = .12***					**Total R**^**2**^adj = .03*				**Total R**^**2**^adj = .04*		

***p≤.001;

**p≤.01;

*p≤.05

^a^ Change in R

^b^ standard beta

^c^ r-square adjusted

Regarding the prediction of attitudes towards inclusion, the inclusion of sociodemographic variables and years of experience in the first step did not improve the prediction of the model (R^2^ = .01, *p* = .63). However, the inclusion of social dominance and opposition to equality in the second step significantly increased the explained variance explained by 11% (R^2^ = .11, p≤. 001). Finally, the inclusion of emotional and cognitive empathy in the third step increased the explained variance in attitudes towards inclusion (R^2^ = .03, *p* = .01). In the last step, the set of variables explained 12% of the variance in attitudes towards inclusion (R _adj_^2^ = .12, p≤.001). Based on observations of each of the variables, social dominance (β = -.18, *p = 01*) was a negative predictor and emotional empathy (*β* = .19, *p* = .01) and opposition to equality (β = .21, *p*≤.001) were positive predictors of attitudes towards inclusion.

Regarding the prediction of sentiments towards inclusive education, in the first step, the inclusion of the sociodemographic variables and years of experience did not significantly increase the explained variance (R^2^ = .01, *p* = .61). In the second step, the addition of social dominance and opposition to equality significantly increased the explained variance by 5% (R^2^ = .05, *p* ≤.01). However, in the last step, empathy did not increase the explained variance (R^2^ = .01, *p* = .90). In this last step, the set of variables explained 32% of the variance in sentiments towards inclusion (R _adj_^2^ = .03, *p* = .03); thus, social dominance was the only variable that showed a significant beta coefficient (β = -. 22, p≤.001) for the positive prediction of sentiments towards inclusive education.

Finally, regarding the prediction of concerns about inclusive education, the inclusion of sociodemographic variables and years of experience in the first step did not improve the prediction of the model (R^2^ = .03, *p* = .08). The inclusion of social dominance and opposition to equality in the second step improved the prediction of the model by 3% (R^2^ = .03, p≤.01); however, the inclusion of cognitive and emotional empathy in the third step did not increase the explained variance (R^2^ = .01, *p* = .39). In the last step, the set of variables explained 4% of the variance in inclusion concerns (R _adj_^2^ = .04, p = .02). It was observed that the only significant beta positive coefficient was for social dominance (β = .19, *p* = .01).

### Fuzzy-set qualitative comparative analysis (QCA)

#### Necessary condition analysis

Based on the results obtained (Table 4), it seems that there were no necessary conditions for the occurrence or non-occurrence of the SACIE-R dimensions (sentiments, attitudes, and concerns) because, in all cases, the consistency was less than .90 [58]

**Table 4:** Necessary analysis for inclusive education dimensions (SACIE-R).

	Attitudes		~^a^Attitudes		Sentiments		~^a^Sentiments		Concerns		~^a^Concerns	
	Cons^b^	Cov^c^	Cons	Cov	Cons	Cov	Cons	Cov	Cons	Cov	Cons	Cov
Woman	.65	.50	.63	.50	.63	.48	.66	.52	.67	.48	.62	.52
Man	.35	.48	.37	.52	.37	.51	.34	.49	.33	.44	.38	.56
Older	.59	.59	.60	.62	.60	.60	.60	.63	.57	.54	.61	.66
Younger	.62	.60	.60	.60	.62	.60	.61	.61	.64	.59	.58	.61
High TE^d^	.60	.58	.62	.62	.64	.62	.61	.61	.59	.54	.63	.67
Low TE	.61	.61	.58	.60	.60	.60	.62	.64	.64	.60	.56	.61
High SD^e^	.45	.54	.58	.71	.60	.71	.45	.55	.60	.68	.45	.58
Low SD	.76	.64	.62	.54	.62	.52	.77	.67	.63	.50	.75	.68
High EO^f^	.67	.66	.53	.54	.59	.58	.62	.64	.58	.54	.62	.66
Low EO	.53	.52	.67	.68	.63	.61	.59	.60	.64	.59	.57	.60
High EE^g^	.61	.66	.51	.57	.57	.61	.58	.66	.58	.60	.57	.67
Low EE	.61	.55	.70	.65	.69	.61	.65	.61	.68	.58	.57	.67
High CE^h^	.59	.61	.58	.62	.56	.59	.61	.66	.60	.57	.56	.69
Low CE	.64	.59	.64	.61	.67	.62	.62	.60	.68	.60	.61	.62

^a^ absence of condition

^b^ consistency

^c^ coverage

^d^ teaching experience

^e^ social dominance

^f^ equality opposition

^g^ emotional empathy

^h^ cognitive empathy

#### Sufficiency analysis

In the sufficiency analyses, the resulting models for each of the dimensions yielded the following results based on the premise that in QCA, a model is informative when the consistency is around or above .75 [56].

Regarding the prediction of attitudes (Table 5), 17 paths were observed that explained 59% of the cases of high levels of positive attitudes towards inclusion (overall consistency = .76; overall coverage = .59). Of these, the three most relevant pathways for explaining high levels of positive attitudes towards inclusion are listed below. The first path was the interaction of high cognitive empathy, high opposition to equality, low social dominance, low work experience, and young age (raw coverage = .25; consistency = .83), which explained 25% of the cases of high levels of positive attitudes towards inclusion. The second path was the interaction between high emotional empathy, low social dominance, older age, female sex, and high work experience (raw coverage = .22; consistency = .82), which explained 22% of cases. Finally, the third path was the combination of high emotional and cognitive empathy, high opposition to equality, young age, female sex, and low work experience (raw coverage = .17; consistency = .82), which explained 17% of cases. Regarding the prediction of low levels of positive attitudes towards inclusion, nine paths were observed that explained 58% of the cases of low levels of positive attitudes towards inclusive education (overall consistency = .79; overall coverage = .58). The most relevant pathway or combination that predicted low levels of positive attitudes towards inclusion was the result of the interaction between low cognitive empathy and emotional empathy, high social dominance orientation, and young age (raw coverage = .25, consistency = .84), which explained 25% of the cases of low levels of positive attitudes towards inclusive education. The second pathway was the interaction between low emotional empathy, low opposition to equality, young age and female sex (raw coverage = .22; consistency = .86), which explained 22% of cases. Finally, the third pathway was the combination of low cognitive empathy, high emotional empathy, high social dominance, and older age (raw coverage = .22; consistency = .91), which explained 22% of cases.

**Table 5:** Summary of the three main sufficient conditions for the intermediate solution of dimensions to SACIE-R.

*Frequency cut-off*: *1*	Attitudes *Consistency cut-off*: .*82*			~^a^Attitudes *Consistency cut-off*: .*84*			Sentiments *Consistency cut-off*: .*86*			~Sentiments *Consistency cut-off*: .*85*			Concerns *Consistency cut-off*: .*85*			~Concerns *Consistency cut-off*: .*88*		
	1	2	3	1	2	3	1	2	3	1	2	3	1	2	3	1	2	3
Woman		P^b^	P		P					P		P	P	P			P	A
Older	A^c^	P	A	A	A	P		P	P		A							
TE^d^	A	P	A					P		P		P		P	A	A	P	
SD^e^	A	A		P		P		P	P	A	A			P	P	A	A	A
EO^f^	P		P		A		A		P			P	A				P	A
EC^g^	P		P	A		P	A	P			P	A	A		A	P		
EE^h^		P	P	A	A	A	P		A	P	A				P	P	P	
Raw coverage	.25	.22	.17	.25	.22	.22	.29	.25	.22	.24	.24	.21	.33	.26	.24	.27	.20	.19
Unique coverage	.01	.01	.01	.01	.04	.03	.04	.04	.03	.07	.03	.04	.07	.04	.01	.02	.03	.04
Consistency	.83	.82	.82	.84	.86	.91	.81	.86	.84	.83	.86	.83	.80	.83	.86	.87	.87	.82
**Overall solution consistency**			**.76**			**.79**			**.77**			**.80**			**.78**			**.80**
**Overall solution coverage**			**.59**			**.58**			**.59**			**.57**			**.60**			**.54**

^a^ absence of condition

^b^ presence of condition

^c^ absence of condition.

^d^ teaching experience

^e^ social dominance

^f^ equality opposition

^g^ cognitive empathy

^h^ emotional empathy

Expected vector for attitudes and sentiments: 1.1.0.1.1 (0: absent; 1: present); Expected vector for concerns: 0.0.1.1.0.0

Expected vector for ~ attitudes and ~sentiments: 0.0.1.0.0.0; Expected vector for ~concerns 1.1.0.0.1 1. Using the format of. Fiss[71]

In the prediction of high levels of sentiments towards inclusive education, ten paths were observed that explained 59% of the cases of high levels of sentiments towards inclusive education (with this dimension understood in a negative sense) (overall consistency = .77; overall coverage = .59). The most relevant path or combination was the result of the combination of low cognitive empathy, high emotional empathy and low opposition to equality (raw coverage = .29, consistency = .81), which explained 29% of cases of high levels of sentiments towards inclusion. The second path was the interaction between high cognitive empathy, high social dominance, older age, and high work experience (raw coverage = .25; consistency = .86), which explained 25% of cases. Finally, the third pathway was the combination of low emotional empathy, high social dominance and opposition to equality and older age (raw coverage = .22; consistency = .84), which explained 22% of cases.

Furthermore, with regard to the observation of low levels of sentiments towards inclusion, nine paths were observed that explained 57% of the cases of low levels of sentiments (overall consistency = .80; overall coverage = .57). The most relevant path or combination that predicted low levels of sentiments towards inclusive education was the result of the interaction of high emotional empathy, low social dominance, low work experience, and older age (raw coverage = .24, consistency = .83), which explained 24% of the cases of low levels of sentiments towards inclusive education. The second path was the interaction between low emotional empathy, high cognitive empathy, low social dominance, and young age (raw coverage = .24; consistency = .86), which explained 24% of cases. Finally, the third pathway was the combination of low cognitive empathy, high opposition to equality, young age, and high work experience (raw coverage = .22; consistency = .83), which explained 22% of cases.

Likewise, in the prediction of high levels of concerns about inclusive education, ten paths were observed that explained 60% of the cases with high levels of concerns about inclusion (with this dimension understood in a negative sense) (overall consistency = .78; overall coverage = .60). The most relevant path or combination to predict high levels of concerns about inclusive education was the result of the interaction between low cognitive empathy, low opposition to equality, and female sex (raw coverage = .33, consistency = .80), which explained 33% of cases of high levels of concerns about inclusion. The second pathway was the interaction between high social dominance, female sex, and high work experience (raw coverage = .26; consistency = .83), which explained 26% of cases. The third combination was high emotional empathy, low cognitive empathy, high social dominance, and low work experience (raw coverage = .24; consistency = .86), which explained 24% of cases.

With regard to the observation of low levels of concerns about inclusive education, nine paths were observed that explained 54% of the cases of low levels of concerns (raw coverage = .54, consistency = .80). The most relevant pathway or combination that predicted concerns about inclusive education was the result of the interaction of high emotional empathy, low cognitive empathy, high social dominance, and low work experience (raw coverage = .27, consistency = .87), which explained 27% of cases of low sentiments about inclusive education. The second path was the interaction among high emotional empathy, low social dominance, high opposition to equality, high work experience, and female sex (raw coverage = .20, consistency = .87), which explained 20% of cases. Finally, the third path was the combination of low opposition to equality and social dominance and male sex (raw coverage = .19; consistency = .82), which explained 19% of cases.

## Discussion

Based on the results obtained from the HRMs concerning the prediction of attitudes towards inclusion, three main predictor variables were revealed: both emotional empathy and opposition to equality positively predicted attitudes, whereas social dominance negatively predicted attitudes. Regarding the prediction of concerns about and sentiments towards inclusion, it seems that social dominance was the only significant positive predictor.

Regarding the QCA models, in general, it seems that none of the conditions were necessary for high or low levels of attitudes, sentiments and concerns regarding inclusive education. In terms of the sufficiency analyses, it appears that to obtain high levels of positive attitudes towards inclusion, a combination of high cognitive and emotional empathy, low social dominance and female sex is necessary. On the other hand, more positive sentiments related to inclusion (indicated by low levels of sentiments in the results because this dimension was measured in a negative sense) were observed in subjects who exhibited low social dominance, who were women, and who had many years of experience. Finally, with regard to greater concerns about inclusive education (indicated by low levels of concerns in the results because this dimension was measured in a negative sense), it was observed that low social dominance and high emotional empathy were the main variables that explained the combinations.

Regarding the study hypotheses, the HRM results seem to provide evidence that partially supports H1 because the SDO significantly negatively predicted attitudes and positively predicted opposition to equality. The rest of the dimensions also confirmed the hypothesis. Social dominance significantly positively predicted concerns and sentiments; nevertheless, opposition to equality was not a significant predictor of these outcomes, which was supported by the results of the QCA. In general, the results obtained seem to be in line with previous studies[7–9]. H2 was also partially supported because emotional empathy only positively predicted attitudes towards educational inclusion. Both emotional and cognitive empathy were important for the QCA models of attitudes and concerns about inclusion, which also seems to be in line with the existing literature[25,26]. The dimensions of attitudes and sentiments towards inclusion correspond with the cognitive aspects of Loreman, Earle, and Forlin’s model[51], while the concerns dimension corresponds to the emotional aspects of attitudes. For this reason, it seems logical that attitudes and sentiments are more closely related to cognitive empathy, whereas the concerns dimension is more closely related to emotional empathy. Finally, neither the sociodemographic variables (age and sex) nor experience predicted inclusive attitudes towards education, contrary to previous studies[13,47,48]. This would lead to the rejection of H3, H4, and H5, which hypothesised that sex, age, and experience would predict attitudes towards inclusion. However, considering the results of the QCA, these variables were important in predicting high or low levels of positive attitudes towards inclusion (sentiments, attitudes, and concerns), which would support the hypotheses, in line with the existing literature[13,47,48].

Promoting inclusive education is a priority objective in current society. Studies indicate that the most important aspect for improving inclusion is inclusive attitudes[5,12,16,17,21]. Among the various studies that have analysed the topic, some research has focused on analysing variables such as empathy[11,18,26,30], while other studies have focused on demographic variables such as sex or age but have not taken into account other types of variables, such as SDO[48]. There is a lack of studies that have considered and analysed this last variable in depth, and many have not analysed the role of all the variables overall. Likewise, most of the studies in psychology have focused on the use of linear models, ignoring other types of methodologies[30]. The combined use of different methodologies, such as HRM and QCA, allows for in-depth study of the relationship between the variables under study. In a comparison of both methodologies, it is observed that the QCA models had greater predictive value as they allowed us to combine different conditions for an increase in predictive value of approximately 60% compared to that of the HRM models. Likewise, the QCA models allowed us to consider the importance of variables that, although they were not significant in isolation, were significant in interaction with other variables or conditions, such as sociodemographic variables (age and sex) and previous experience and empathy. The importance of these variables would have been overlooked if we had only used HRMs. Finally, it is observed that the prediction of high levels for each of the subscales did not depend on the same factors or conditions as the prediction of low levels, which could not have been verified from an analysis based on linear models.

Based on the above and given that regression models and QCA models are useful for the accomplishment of different objectives, we suggest that instead of focusing on one type of model in research, we should advocate complementarity, i.e., the simultaneous use of both techniques as indicated in previous studies[65–69]. As previously stated, there appear to be no studies that have analysed the impact of empathy, social dominance orientation, age, sex, and experience in the same study, nor have we been able to find any study that considers any of these variables in isolation or together; all studies we identified used non-linear models, such as QCA models. The complementary use of methodologies would allow analysis of the relationships between variables that have been studied separately but not jointly. This is relevant for practice because it provides us with knowledge about what skills can be trained through intervention programmes with teachers to ensure inclusive education.

Despite the contributions made by the present study, the research is not without limitations. One of the main limitations is related to the sample of the study. The sampling procedures were not probabilistic, and the geographical location of the study, which was based solely on teachers from schools in the Valencian Community, made it difficult to generalise the results. Another limitation is related to the use of questionnaires. Although questionnaires are a common research tool, they can introduce social desirability biases. Future research should overcome these limitations by using probability sampling procedures, broadening the geographical area and cultural contexts from which the study sample is recruited, and using other types of measures that are objective or that consider not only behavioural dispositions, intentions, or attitudes but also teachers’ own behaviour in the classroom.

Despite the limitations, this study is of special interest because it offers guidelines that should be taken into account by public and private entities to promote school inclusion, such as promoting empathy in teachers, developing programmes to reduce social dominance orientation, and considering differential intervention programmes depending on the age, experience, or sex of the participants, as suggested by the results of the QCA. Likewise, from a scientific point of view, this study allowed the analysis of variables that have previously not been considered in the same study to predict teachers’ attitudes towards inclusion, and it offers evidence on the usefulness of combining two differential but complementary methodologies, i.e., hierarchical regression modelling and the QCA. The latter technique is infrequently used in the context of social psychology or psychology in general.

In conclusion, it seems that adequate emotional skills, such as empathy and low social dominance orientation, high opposition to equality, and female sex have positive impacts on teachers’ attitudes towards inclusive education. The use of two complementary methodologies, hierarchical regression modelling and QCA, provides more information when analysing the results. The comparison of new methodologies allows horizons to be broadened at the methodological level by providing new tools that can be applied in various contexts. The relevance of the study results to practice allows professionals involved in inclusive education to determine which skills can be developed through training and to become empowered so that teachers and society in general can meet the challenge of promoting more inclusive education.

## Supporting information

S1 Data set Inclusiveeduccation_HRM. (DAT)

S2 Data set Inclusiveeducation_QCA. (DAT)
